# Energy cost of running and Achilles tendon stiffness in man and woman trained runners

**DOI:** 10.1002/phy2.178

**Published:** 2013-12-06

**Authors:** Jared R. Fletcher, Ted R. Pfister, Brian R. MacIntosh

**Affiliations:** 1 Human Performance Laboratory, Faculty of Kinesiology, University of Calgary, Calgary, Alberta, Canada

**Keywords:** Allometric scaling, economy of locomotion, oxygen uptake, respiratory exchange ratio, running economy

## Abstract

The energy cost of running (*E*_run_), a key determinant of distance running performance, is influenced by several factors. Although it is important to express *E*_run_ as energy cost, no study has used this approach to compare similarly trained men and women. Furthermore, the relationship between Achilles tendon (AT) stiffness and *E*_run_ has not been compared between men and women. Therefore, our purpose was to determine if sex‐specific differences in *E*_run_ and/or AT stiffness existed. *E*_run_ (kcal kg^−1^ km^−1^) was determined by indirect calorimetry at 75%, 85%, and 95% of the speed at lactate threshold (sLT) on 11 man (mean ± SEM, 35 ± 1 years, 177 ± 1 cm, 78 ± 1 kg, 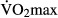
1 = 56 ± 1 mL kg^−1^ min^−1^) and 18 woman (33 ± 1 years, 165 ± 1 cm, 58 ± 1 kg, 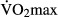
2 = 50 ± 0.3 mL kg^−1^ min^−1^) runners. AT stiffness was measured using ultrasound with dynamometry. Man *E*_run_ was 1.01 ± 0.06, 1.04 ± 0.07, and 1.07 ± 0.07 kcal kg^−1^ km^−1^. Woman *E*_run_ was 1.05 ± 0.10, 1.07 ± 0.09, and 1.09 ± 0.10 kcal kg^−1^ km^−1^. There was no significant sex effect for *E*_run_ or RER, but both increased with speed (*P* < 0.01) expressed relative to sLT. High‐range AT stiffness was 191 ± 5.1 N mm^−1^ for men and 125 ± 5.5 N mm^−1^, for women (*P* < 0.001). The relationship between low‐range AT stiffness and *E*_run_ was significant at all measured speeds for women (*r*^2^ = 0.198, *P* < 0.05), but not for the men. These results indicate that when *E*_run_ is measured at the same relative intensity, there are no sex‐specific differences in *E*_run_ or substrate use. Furthermore, differences in *E*_run_ cannot be explained solely by differences in AT stiffness.

## Abstract (precis)

Here, we show that when energy cost of running is normalized to body mass, at similar relative speeds of running, no sex‐specific differences in substrate use nor in the energy cost of running exist among similarly trained runners. Furthermore, the stiffness of the Achilles tendon (AT) of women is lower than in males, but the relationship between *E*_run_ and AT stiffness is not different between the sexes.

## Introduction

Several studies have been concerned with the main physiological determinants of performance in distance running. These determinants include, among other variables: maximal oxygen uptake (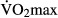
3), fractional utilization of 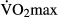
4, the ability to withstand a disturbance in homeostasis, or tolerance, and the energy cost that is required to transport the body over a given distance (Di Prampero et al. 1993). In the case of running, this latter variable is typically defined as a subject's energy cost of running (*E*_run_). In a heterogeneous group of runners with a large range of 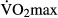
5 values, a strong positive relationship exists between running performance and 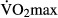
6 (Costill et al. 1973). Among runners who possess similar 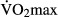
7 values, *E*_run_ becomes a better predictor of running performance than 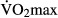
8 alone (Pollock 1977).

Several studies have compared *E*_run_ between men and women and some argue that no difference exists (Pate et al. 1992; Ingham et al. 2008). In contrast, some contest that woman runners are more economical than their man counterparts (Helgerud 1994; Helgerud et al. 2010). Still others argue to the contrary; men are more economical (Daniels and Daniels 1992). Further study of this controversy may allow a better understanding of the fundamentals that affect *E*_run_. As we have previously outlined (Fletcher et al. 2009), appropriate measurement of *E*_run_ should be performed at similar %speed at lactate threshold (sLT) between individuals, in order to ensure that the steady state of 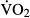
9 is achieved and to minimize differences in substrate use (as reflected by the steady‐state RER) between individuals, knowing that the energy equivalent of 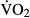
10 changes with RER. It also makes sense to test at a speed that relates to a competition distance, like a 10‐km race (i.e., a similar % sLT). None of the previous studies comparing *E*_run_ of men and women has done this. *E*_run_ should also be expressed as an energy cost per unit distance rather than a 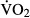
11 to allow comparison between different absolute speeds. Thus, much of the conflicting evidence previously presented regarding *E*_run_ between men and women may result from an inappropriate expression of *E*_run_ itself.

In one of the two studies to date that have compared *E*_run_ in man and woman runners at similar relative intensities (relative to maximal oxygen uptake), *E*_run_ was expressed as mL kg^−0.75^ m^−1^. It was reported that the lighter woman runners were more economical than their heavier man counterparts (Helgerud et al. 2010). In the other study (Tarnopolsky et al. 1990), *E*_run_ was expressed as kcal/kg to run a given distance, and no significant difference between men and women was detected. Furthermore, no study has made direct comparison between similarly trained man and woman runners that have expressed *E*_run_ in terms of energy (kcal or J kg^−1^ m^−1^). Could it be that sex‐specific differences in *E*_run_ may be confounded as a result of differences in allometric scaling for body mass on metabolic rate? As metabolic rate does not increase to the same extent as body mass (Bergh et al. 1991; Rogers et al. 1995), allometric scaling for body mass has been used when comparing *E*_run_ for groups of varied body mass. The allometric scaling relationship is as follows:
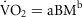
1

where BM is body mass, a is a constant, and b is the scaling exponent. Where the relationship between BM and 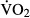
13 is linear, the value of b should be 1.

Therefore, it needs to be confirmed whether the previous use of an allometric scaling factor of b = 0.75 has influenced the comparison of *E*_run_ between sexes, or whether this is simply a function of the cohort tested and/or of the methods used in the determination of *E*_run_. As we have proposed previously, *E*_run_ should be measured at similar intensities expressed relative to sLT for all runners.

It has become apparent in recent years that the muscle‐tendon unit mechanical properties of the lower limbs may be important as determinants of *E*_run_. Specifically, it has been shown that Achilles tendon (AT) stiffness is generally less than optimal and greater stiffness corresponds with a lower energy cost of running (Arampatzis et al. 2006; Lichtwark and Wilson 2008; Fletcher et al. 2010; Kubo et al. 2010). Recently, we have demonstrated that it is a combination of factors, including muscle shortening, shortening velocity, and level of muscle activation that dictate the energy cost of muscle contraction in vivo(Fletcher et al. 2013) and this may help explain the relationship between AT stiffness and *E*_run_.

To date, virtually nothing is known about the muscle‐tendon interactions in woman runners. Woman AT stiffness is generally thought to be lower than the AT stiffness in similarly trained men (Kubo et al. 2003). This may be a result of women having a lower isometric strength. The relationship between strength and AT stiffness is well‐reported (Muraoka et al. 2005). Given the reported relationship between AT stiffness and *E*_run_, it seems logical to hypothesize that *E*_run_ of women would be greater than that of the men. This hypothesis has not been tested directly to date. If *E*_run_ is not different between sexes, then the question exists: why are the ATs of woman runners less stiff than their man counterparts? We hypothesize that this level of AT stiffness is required in the woman runners, running at a slower speed than the men, in order to keep muscle shortening velocity low.

Therefore, the primary purpose of this study was to investigate *E*_run_ and AT stiffness between similarly trained man and woman runners and to determine if sex‐specific differences in *E*_run_ and/or AT mechanical properties exist. In order to do so, and to compare directly with current literature, the use of allometric scaling for body mass was also considered.

## Material and Methods

### Ethical approval

Man (Fletcher et al. 2010) and woman (Ingham et al. 2008) trained runners participated in this study (Table 1). The runners gave their informed written consent to participate in the experimental procedures, which were approved by the University of Calgary Conjoint Health Research Ethics Board.

**Table 1.** Subject characteristics.

Gender	*N*	Age (years)	Height (m)	Mass (kg)	(mL kg^−1^ min^−1^)	sLT (m min^−1^)	VO_2_ at sLT (%VO_2_ max)
Man	11	35.3 ± 0.8	1.77 ± 0.04*	77.6 ± 0.7*	55.5 ± 0.8*	234.1 ± 2.8*	89.3 ± 0.7
Woman	18	32.8 ± 0.9	1.65 ± 0.07	57.9 ± 0.6	49.8 ± 0.6	202.9 ± 2.0	88.3 ± 0.6

Values are mean ± SEM.

* Significantly different (*P* < 0.05), men versus women.

### Experimental protocol

All subjects participated in training for running a minimum of five times per week and none of the subjects had any neuromuscular or musculoskeletal injuries at the time of the study. All subjects were following a similar periodized training plan for either the 10 km or half‐marathon road race distance. Self‐reported estimates of current 10‐km race time (mean ± SD) were 39.67 ± 4.51 min for the men and 47.17 ± 6.07 min for the women. This difference in race time was significant (*P* < 0.002). When compared to the current National records for the 10‐km race distance, the mean man and woman race times were 30.2 ± 7.5% and 32.1 ± 8.4% slower than National record times (http://athletics.ca/page.asp?id=66), respectively, illustrating a similar level of performance in the two groups (*P* = 0.53).

The subjects visited the laboratory on two separate occasions. On the first visit, an incremental exercise test to exhaustion was performed on a treadmill (Woodway Pro, Woodway USA, Waukeshka, WA) to determine the subject's maximal oxygen uptake (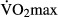
14) and speed associated with the lactate threshold (sLT). Prior to arriving at the lab, subjects were instructed not to consume any food or beverage, other than water, for a minimum of 12 h prior to the testing. They were also asked to refrain from the ingestion of caffeine and to avoid vigorous physical activity for 24 h prior to the testing. The subjects wore cool, loose clothing and their own lightweight running shoes.


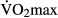
15 and sLT were determined based on methods used previously in our lab (Fletcher et al. 2009, 2010). Following a self‐selected warm‐up of no more than 15 min of running, the subjects began running on a motorized treadmill with zero gradient at ~3 km h^−1^ slower than the subject's self‐reported 10 km race pace. Expired gases were collected by a metabolic cart (Parvomedics Truemax 2400, Salt Lake City, UT) for the determination of 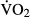
16 (mL kg^−1^ min^−1^) and carbon dioxide output (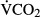
17, mL kg^−1^ min^−1^). The metabolic cart was calibrated before and after each testing session, as described previously by Fletcher et al. (2009). The treadmill speed was increased by 0.48 km h^−1^ every 2 min. After each 2‐min stage, the subjects briefly straddled the belt and a fingertip blood sample was taken for the determination of blood lactate concentration ([BLa^‐^], Lactate Pro). When [BLa^‐^] rose more than 1 mmol/L from the previous sample, the treadmill belt was returned to the previous speed and the gradient was increased 2% every minute until the subject was unwilling to continue.

sLT was defined as the speed at the stage preceding that which elicited a [BLa^‐^] increase in greater than 1 mmol/L. All tests were terminated due to volitional exhaustion. 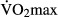
18 was defined as the highest 30‐sec average 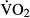
19 during the test and was said to have been reached if there was an increase in 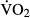
20 no greater than 2 mL∙kg^−1^∙min^−1^with an increase in treadmill gradient. Of the 29 subjects, 18 achieved 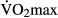
21 based on this criterion. In the other 11 subjects, 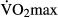
22 was said to have been reached if two of the following occurred: (1) RER greater than 1.15; (2) [BLa^‐^] greater than 8 mmol/L; or (3) subjects reached their age‐predicted maximal heart rate (220 beats min^−1^ − age). All of the remaining 11 subjects achieved 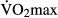
23 based on these criteria.

Between 48 and 72 h following the 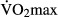
24 testing session, the subjects returned to the laboratory for determination of AT stiffness and *E*_run_. The subjects followed the same pretesting instructions as the first testing session. AT stiffness was determined on the right leg as described previously by Fletcher et al. (2010). The subjects laid prone with their knee at 180° and their ankle at 90°. Before each MVC, the axis of rotation of the dynamometer (Biodex, Medical Systems Inc., Shirley, NY) was carefully aligned with the axis of rotation of the ankle joint. The shank and unshod foot were affixed to the dynamometer using a series of Velcro straps. The subjects performed three isometric ramp maximal voluntary contractions (MVC) plantarflexions. Moment during the MVC was sampled at 100 Hz. The trial eliciting the highest moment was used for analysis.

During each MVC, a 12.5 MHz linear array ultrasound probe (50 mm, Philips Envisor, Philips Healthcare, Eindhoven, Netherlands) was used to visualize the medial gastrocnemius (MG) muscle fascicles, close to the AT. The ultrasound probe was placed on the MG muscle belly, near the myotendinous junction, and secured using a custom‐built apparatus. Ultrasound scans were recorded at 49 Hz. A clear point where a fascicle inserts into the deep aponeurosis was followed throughout the MVC and its displacement was measured using ImageJ, (NIH, Baltimore, MD). This displacement of a fascicle‐aponeurosis junction was considered tendon elongation. An external function generator (B‐K Precision 3010; Dynascan Corp., Chicago, IL) was manually started at the beginning of the MVC and served as a timestamp between image and moment data collection.

### Correction for joint rotation

Despite affixing the ankle joint to the dynamometer tightly with Velcro straps, joint rotation during the MVC is inevitable (Magnusson et al. 2001). This inevitable joint rotation would result in a lower resultant torque and would contribute erroneously to the apparent tendon elongation measured during the contraction (Spoor et al. 1990; Muramatsu et al. 2001). The resultant moment and apparent tendon elongation were corrected for this motion, as described previously (Fletcher et al. 2010). Ankle joint motion during the contraction was imaged at 30 Hz using a portable video camera (Canon GL1, Canon Inc., Tokyo, Japan). Joint angle change was determined by following two to four small dots drawn on the medial aspect of the unshod right foot. From this, ankle joint angle could be calculated throughout the contraction using ImageJ. We assumed that the moment about the ankle resulted in a force perpendicular to the foot. Any change in angle of the foot relative to the Biodex lever will result in an underestimation of the ankle joint moment. To estimate this error, we measured the change in angle of the foot relative to the Biodex lever, and the corrected moments were calculated as:
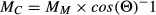
2where M_C_ and M_M_ are the corrected and measured moments, respectively, and θ, the angle of the foot during the MVC. The corrected moments were used for further calculation of plantarflexion force.

The moment arm of the AT was estimated using the tendon travel method (An et al. 1984) under in vivoconditions (Ito et al. 2000; Maganaris 2000). The displacement of a fascicle‐aponeurosis cross‐point (d_L_, mm) caused by passively rotating the ankle at 10° sec^−1^ from 5º of dorsiflexion to 5º of plantarflexion (d_θ_, rad) was measured. The AT moment arm was calculated as the ratio d_L_/d_θ_ (mm rad^−1^). Triceps surae force was calculated by dividing the ankle joint moment by the estimated AT moment arm.

AT stiffness was determined by fitting the Force (*F*)‐elongation (*d*_L_) data to a quadratic regression equation using equation:
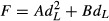
3

Where A and B are constants. AT stiffness was calculated as the slope of the *F*‐*d*_L_ curve at three different force ranges: 25–45% (low‐range stiffness), 30–70% (mid‐range stiffness), and 50–100% (high‐range stiffness) of MVC force (Fletcher et al. 2010). These force ranges were chosen because the lower force ranges may be more similar to forces experienced by the AT during submaximal running (Scott and Winter 1990; Giddings et al. 2000).

After a 10‐min warm‐up at 8 km h^−1^ for the women and 9.6 km h^−1^ for the men, the subjects ran at 75, 85, and 95% sLT for 5 min each, with a 5‐min standing rest period between speeds. [BLa^‐^] was determined immediately prior to and following each speed. *E*_run_ was calculated as the O_2_ cost to cover a given distance (mL O_2_ kg^−1^ km^−1^) as well as the energy cost of running a given distance (kcal kg^−1^ km^−1^), as described previously (Fletcher et al. 2009). The steady‐state 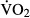
27, defined as the average 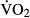
28 over the final 2 min of each stage, was used to calculate *E*_run_. In all cases, the 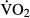
29 over the final 2 min of each stage did not differ more than 2 mL kg^−1^ min^−1^.

### Statistics

Values are presented as mean ± SEM, unless otherwise indicated. A two‐way analysis of variance (ANOVA) (gender, speed) with repeated measures (speed) was used to test for differences in *E*_run_, and for stiffness (repeated measures, sex by force range).When there was no significant interaction and a significant main effect was found, Tukey's post hoc test was used to detect significant differences between the three speeds. Pearson Product‐moment correlation coefficients and simple linear regression were used to evaluate the relationship between BM and metabolic rate. Correlation and linear regression analysis were also used to evaluate the relationship between *E*_run_ and AT stiffness by sex. Sidak's multiple comparison tests were used to correct for more than one linear regression analysis. All analyses were performed using GraphPad Prism version 6.01 for Windows (GraphPad Software, La Jolla, CA, www.graphpad.com). Statistical significance was considered *P* < 0.05.

## Results

Subject characteristics are listed in Table 1. Height and mass were significantly greater in the men compared to the women (*P* < 0.05). The men also had a significantly greater sLT and relative 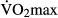
30. The intensity at which sLT occurred (expressed relative to 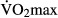
31) was not significantly different between sexes, indicating a similar level of training in these two groups.

**Figure 1.**
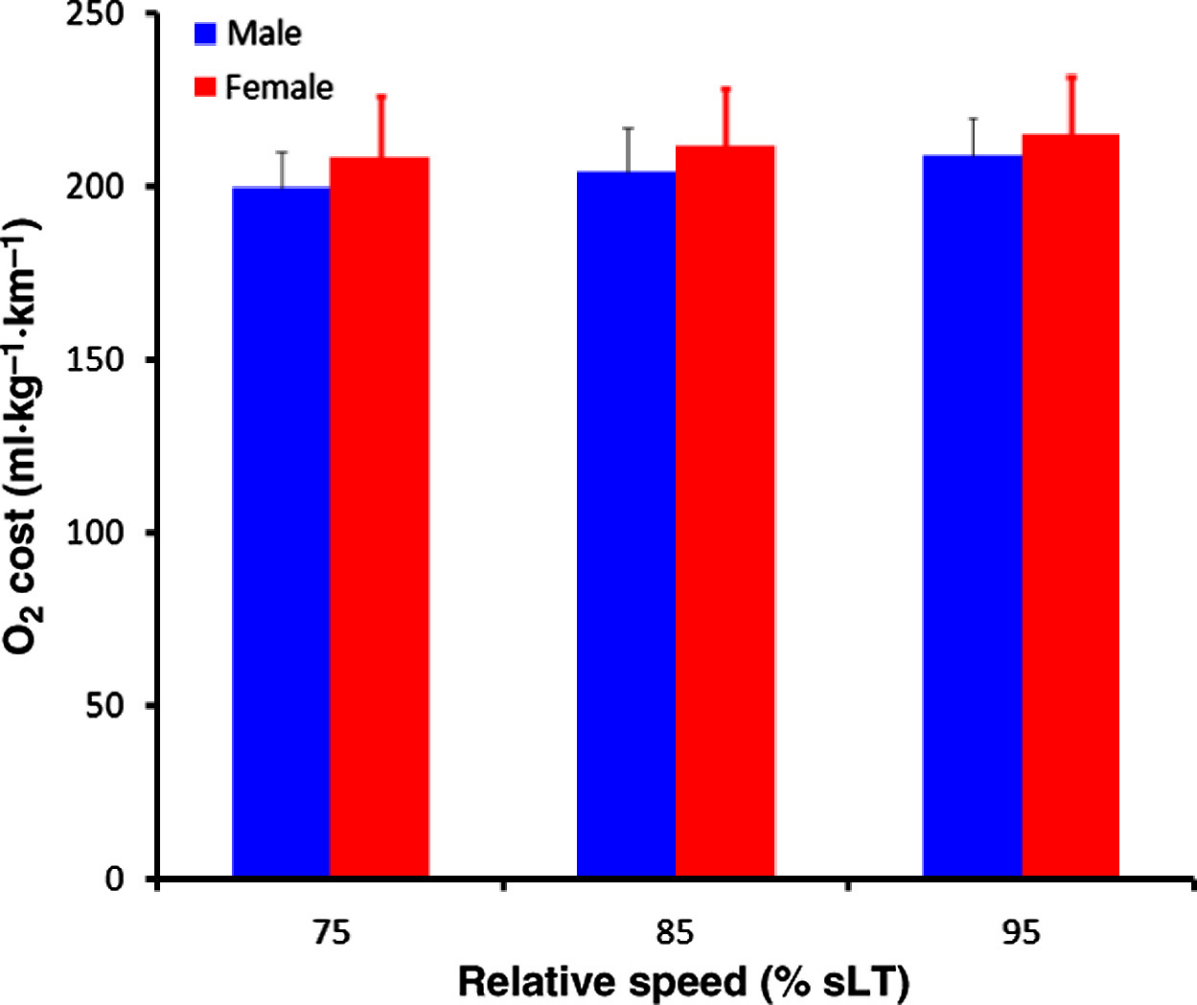
Oxygen cost at the three measured relative speeds in both men and women. Speed is expressed relative to the speed at lactate threshold (sLT). Vertical bars represent SD.

Two‐way repeated measures ANOVA revealed no significant sex by speed interaction (*P* = 0.48) and no differences in O_2_ cost (mL kg^−1^ km^−1^) between sexes (Table 2); however O_2_ cost increased significantly with speed (*P* < 0.0001, Fig. 1). Similarly, there was no significant sex by speed interaction for RER and RER was not significantly different between sexes (*P* = 0.59). RER increased significantly as a function of relative speed (*P* < 0.0001). Consistent with these observations, for O_2_ cost and RER, there was no significant difference in *E*_run_ between sexes (Table 2). *E*_run_ also increased significantly with increasing relative speed (*P* < 0.0001).

**Table 2.** Running economy and RER.

Gender	*N*	O_2_ cost (mL kg^−1^ km^−1^)			RER			Energy cost (kcal kg^−1^ km^−1^)		
sLT		75%	85%	95%	75%	85%	95%	75%	85%	95%
Man	11	200 ± 11	204 ± 13	209 ± 11	0.91 ± 0.03	0.93 ± 0.03	0.97 ± 0.03	1.01 ± 0.06	1.04 ± 0.07	1.07 ± 0.07
Woman	18	209 ± 18	212 ± 17	215 ± 17	0.90 ± 0.03	0.92 ± 0.03	0.96 ± 0.03	1.05 ± 0.10	1.07 ± 0.09	1.09 ± 0.10

Values are mean ± standard deviation.

The use of allometric scaling to BM^−0.75^ did not reduce the interindividual variability in either O_2_ cost or *E*_run_. Furthermore, when our data were scaled to BM^−0.75^, there was still no sex by speed interaction in *E*_run_ (*P* = 0.39) and no main effect for sex (*P* = 0.30).

When man and woman data were combined, there was a significant positive relationship between 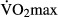
32 and sLT (*r*^2^ = 0.568, *P* < 0.0001), suggesting that runners with the highest 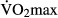
33 also possessed a high sLT. There was no relationship between 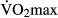
34 and *E*_run_ at any of the measured speeds (*P* = 0.468, 0.790, and 0.983 at 75, 85, and 95% sLT, respectively).

**Figure 2.**
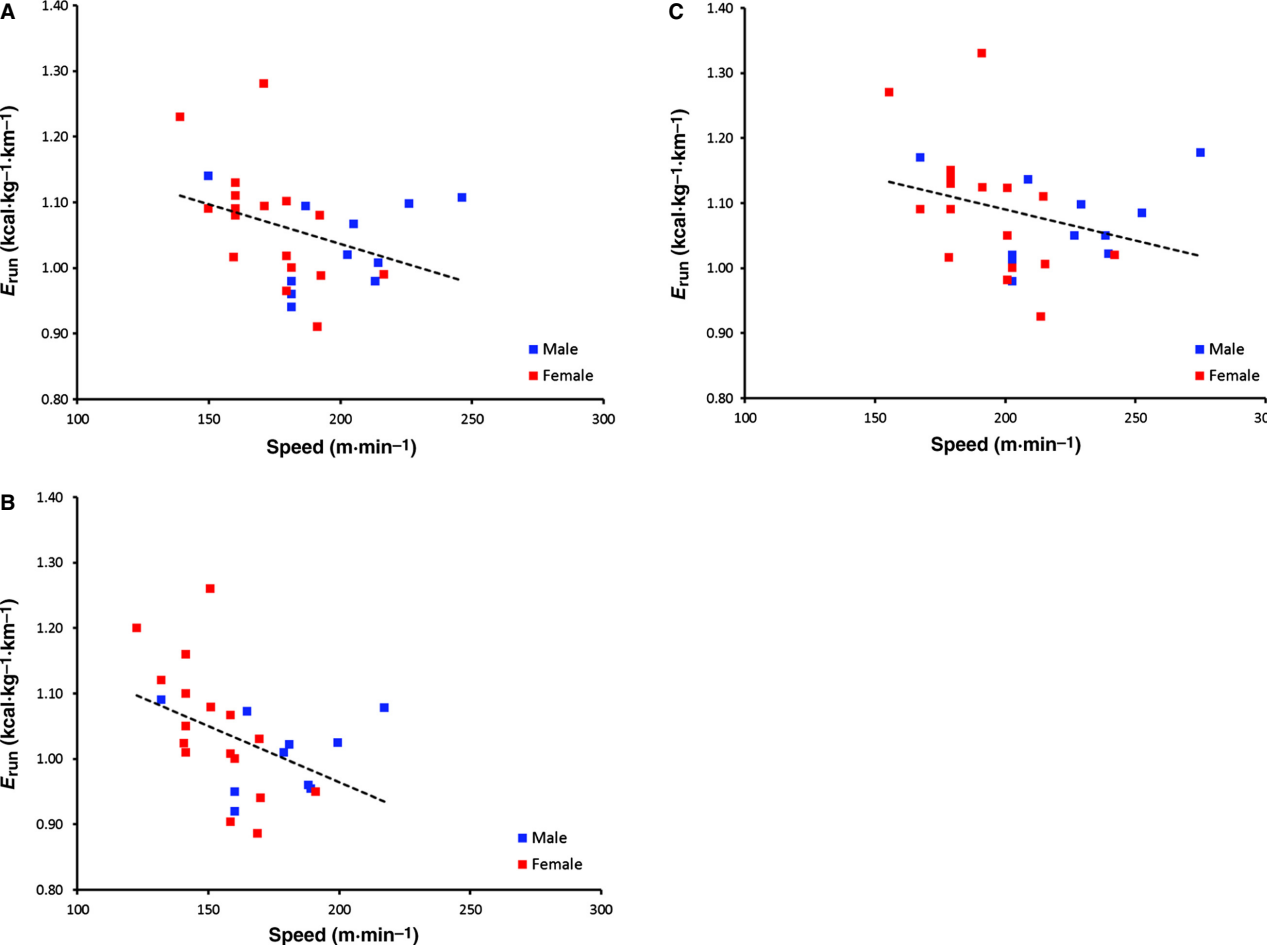
*E*_run_ as a function of absolute running speed. Energy of running was measured at 75% (A), 85% (B), and 95% (C) of sLT.

Figure 2 shows the relationship between *E*_run_ and absolute speed during the *E*_run_ tests. *E*_run_ decreased significantly with increases in absolute speed at 75% sLT (*r*^2^ = 0.197, *P* < 0.02), suggesting the better runners (i.e., the runners with the highest sLT) had a lower EC of running. This relationship did not reach a significant level for the other two relative speeds (*r*^2^ = 0.128, *P* = 0.056 at 85% sLT; *r*^2^ = 0.09, *P* = 0.086 at 95% sLT), suggesting that runners with a high sLT were no more economical than the runners with a low sLT at high relative speeds. The *E*_run_**‐**speed relationship was not affected by substrate use, as no relationship between RER and absolute speed existed (*r*^2^ < 0.05, *P* > 0.238 across all measured relative speeds).

### Tendon mechanical properties

Tendon mechanical properties for both men and women are shown in Table 3. AT stiffness of men was significantly greater than the AT stiffness of the women regardless of force range evaluated (*P* < 0.001). There was also a significant, positive relationship between AT stiffness and body mass for all subjects (*r*^2^ = 0.295, *P* = 0.002).

**Table 3.** Tendon mechanical properties for men and women.

Gender	*N*	AT stiffness (N mm^−1^)		
Force range		25–45%	30–70%	50–100%
Man	11	164 ± 8*	175 ± 6*	191 ± 5*
Woman	18	97 ± 4	108 ± 5	125 ± 5

Values are mean ± standard error of the mean.

* Significantly different (*P* < 0.05), men versus women.

**Figure 3.**
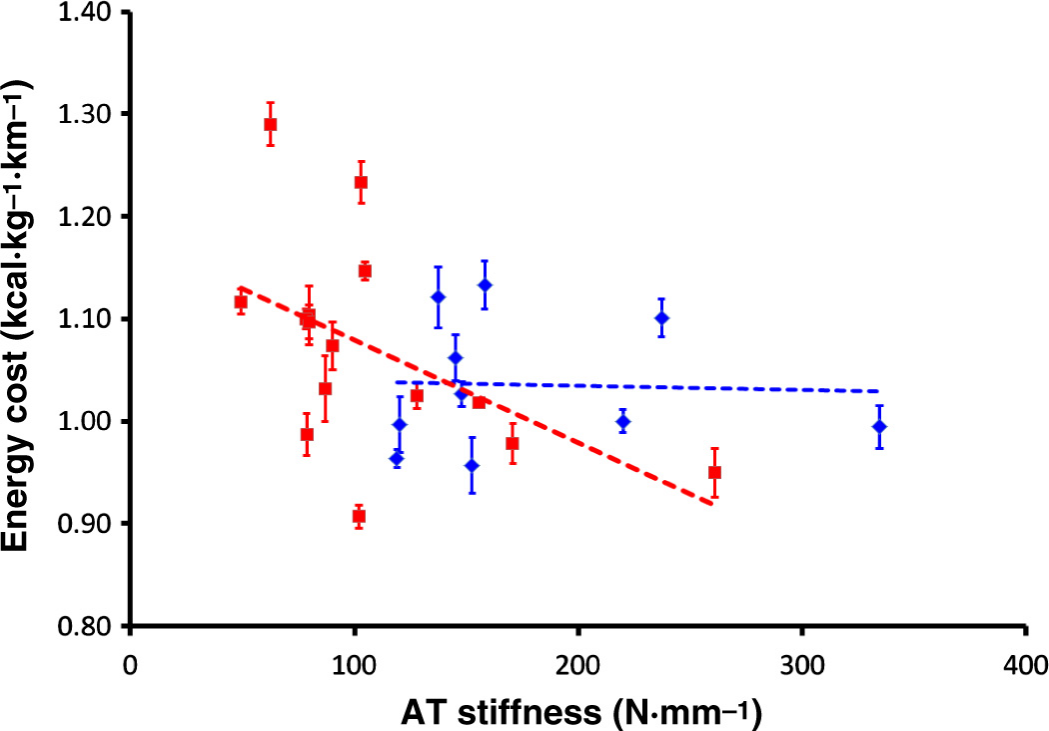
The relationship between Achilles tendon (AT) stiffness and the energy cost of running for men and women. Open and closed dots represent each man and woman subject, respectively. *E*_run_ is represented as mean ± SD for all measured speeds. AT stiffness is shown as the stiffness representing the slope from 50 to 100% MVC of the force‐elongation curve for each subject. Solid and dashed lines represent the linear regression for woman and man subjects, respectively.

Figure 3 shows the relationship between AT stiffness at the highest force range and *E*_run_ in both men and women. There were no significant relationships between AT stiffness and the *E*_run_ at any force range in the men; however in the women, this relationship was significant at all force ranges at 75% sLT (corrected *P* < 0.05), at the lowest force range at 85% sLT (*P* < 0.05), and at the two lowest force ranges at 95% sLT (*P* < 0.05). The relationships at the other speeds and force ranges approached statistical significance in the women (*P* = 0.054 to *P* = 0.084). When both man and woman data were combined, and when AT stiffness was scaled to body mass, the relationship between AT stiffness and *E*_run_ was significant at all measured speeds (*r*^2^ = 0.158–0.191, *P* < 0.033).

## Discussion

The main findings of this study were threefold. First, *E*_run_ did not differ between sexes. This was true when *E*_run_ was expressed either as the energy cost (kcal kg^−1^ km^−1^) or as an O_2_ cost (mL O_2_ kg^−1^ km^−1^). Second, expressing the energy cost relative to BM^−0.75^ did not change this conclusion. Finally, relationships existed between AT stiffness and *E*_run_ in the woman runners and between AT stiffness and body mass in all runners.

We are aware of only one study to date in which *E*_run_ of men and women were reported at similar relative intensities (Helgerud et al. 2010). These authors reported that women had a lower O_2_ cost than men. However, in that study, O_2_ cost was scaled to BM^−0.75^. We observed no differences in O_2_ cost nor in *E*_run_ between the sexes when we scaled O_2_ cost to BM^−0.75^. Another difference between this study and Helgerud et al. (2010) is that our subjects ran on the treadmill at a level grade, while subjects in the Helgerud study ran on the treadmill with a grade of 1.5%. These latter authors report mean O_2_ costs of 670–685 and 753–755 mL kg^−0.75^ km^−1^ at speeds near the sLT for women and men, respectively. We have calculated the mean O_2_ costs of our runners to be approximately 593 and 621 mL kg^−0.75^ km^−1^ at 95% sLT. It seems unlikely that the difference in oxygen cost between our study and that of Helgerud et al. (2010) can be accounted for by this methodological difference, but this methodological difference may contribute to the different results for the between sex comparison.

It seems possible that the methodological difference (slope of the treadmill), coupled with the normalization to 0.75 of body mass are contributing factors for these discrepant results. For example, using the average mass of men and women from the study by Helgerud et al. (2010) it can be calculated that O_2_ cost expressed per kg would have been 254 and 245 mL kg^−1^ km^−1^ for men and women, respectively. This represents a difference of 3.7%, whereas the reported values (752 and 686 mL kg^−1^ km^−0.75^), respectively, differ by 9.6%. This larger difference may have allowed reaching statistical significance. The higher values associated with running on a slope of 1.5% coupled with the allometric scaling may be the main reasons for the finding of a significant difference.

However, it remains unclear whether other factors (apart from the increased work associate with greater BM of the men and expression of O_2_ cost relative to BM^0.75^) may have affected these reported differences. Although it has been found that running on a treadmill with 1% slope more accurately reflects the O_2_ cost of running over ground than running on a treadmill at zero slope (Jones and Doust 1996), the fundamental factors dictating energy cost of running on a flat surface are different from those factors dictating energy cost of running up a slope. Running up a slope at increasing speed will increase the energy cost of running in proportion to body mass, whereas running over ground at increasing speed increases energy cost of running in proportion to frontal surface area and drag coefficient. This study demonstrates, however, that when running on a treadmill with zero gradient, the O_2_ cost of running does not differ between men and women of different body mass.

We have previously shown that in a group of highly trained runners, those runners with a higher sLT have a lower *E*_run_ (Fletcher et al. 2009). This phenomenon is also demonstrated here in lesser trained runners and, for the first time in woman runners (Fig. 2). Our results are consistent with the findings of Pollock (Pollock 1977), who suggest that runners with the lowest *E*_run_ are associated with the fastest running performance. However, in this study, the relationship between *E*_run_ and sLT was not statistically significant at the highest speeds tested (*P* = 0.056 and *P* = 0.086). *E*_run_ is influenced by a variety of factors, and while it is generally accepted that better distance runners are more economical when O_2_ cost is measured at an absolute speed, this is not necessarily the case when *E*_run_ is presented at similar relative intensities. Differences in O_2_ cost at a given speed between individuals are likely a result of the runners being tested at different relative speeds, and it is clear from the current results that *E*_run_ increases with relative intensity. Thus, faster runners running at a given absolute speed are probably running at a lower relative intensity than the slow runners. It seems logical to compare runners at the speed they would be competing in a long distance run. Thus, in order to elucidate any differences in *E*_run_ between men and women, *E*_run_ should be measured at the same relative intensity.

It could be argued that the current sample size is not sufficiently large to detect a difference between sexes in either RER or *E*_run_ and this is, in fact, true. To detect a between‐group difference in RER of 0.03 at a given% sLT, it was estimated that a sample size of >140 per group would be required. Also, given our current data, between 63 and 252 runners per group would be needed to detect a difference in *E*_run_ of the magnitude presented in Table 2 at the measured speeds. The magnitude of difference would be in the order of 3% and if the current results prevailed, women would have the higher *E*_run_.

A secondary purpose of this study was to evaluate the relationship between AT stiffness and *E*_run_ in both man and woman runners. It has been shown previously that a stiff AT is associated with a lower *E*_run_ (Arampatzis et al. 2006; Fletcher et al. 2010). Furthermore, changes in AT stiffness are associated with changes in *E*_run_ (Fletcher et al. 2010), supporting that this is likely a cause and effect relationship. Here, we show a similar stiffness‐*E*_run_ relationship, but only in the women and not in the men, and only when AT stiffness was measured at the lowest force ranges. Furthermore, no clear demarcation between the man and woman data is visible in this relationship. The possibility exists that the small range of *E*_run_ values and low *n* in the men precludes any significant relationship between *E*_run_ and stiffness to be shown.

However, understanding how and why changes in AT stiffness are associated with changes in *E*_run_ are difficult to elucidate. We speculate that AT stiffness is finely tuned in order to minimize the shortening of the muscle in series with it. This reduces the muscle energy cost (Fletcher et al. 2013).

It has been previously shown that energy cost is related to the amount and/or velocity of muscle shortening (Askew and Marsh 1998,) as well as the level of muscle activation, which is necessarily higher to achieve a given force when velocity of shortening is greater (Fletcher et al. 2013). During the stance phase of running, the AT will stretch and subsequent passive recoil of the AT will contribute to positive mechanical work of the muscle‐tendon unit at the end of the stance phase (Biewener and Roberts 2000) decreasing the need for work contributed by the fascicles, which can remain near isometric (Hof et al. 2002; Ishikawa et al. 2007; Lichtwark et al. 2007). For the same load or force exerted by a muscle, a stiffer tendon reduces the amount of energy storage and return, but minimizes the energy cost of the muscle contraction as it reduces the amount of muscle shortening required to effect joint rotation, thereby reducing the metabolic cost.

Ultimately, optimal AT stiffness is the stiffness which allows the maximal contribution of positive mechanical work by the tendon while keeping the muscle fascicle shortening velocity low during muscle activation. This keeps active muscle volume to a minimum (Barclay et al. 2008). It should be kept in mind, however, that we have only examined the mechanical properties of the tendon of one muscle group (the triceps surae), which does not solely dictate the *E*_run_. Furthermore, *E*_run_ is influenced by a variety of factors (Saunders et al. 2004), tendon mechanical properties being just one of these.

In conclusion, the main finding of this study was that when energy cost of running is normalized to body mass, at similar relative speeds of running, no sex‐specific differences in substrate use nor *E*_run_ exist among similarly trained runners. Furthermore, the stiffness of the AT of women is lower than in men, but the relationship between *E*_run_ and AT stiffness is not different between the sexes.

## Acknowledgments

The authors thank the subjects for their time and effort in completing the experimental protocol. J. R. F. was supported by NSERC Canada. T. R. P. was supported by the Prize for Undergraduate Research Excellence (PURE), University of Calgary.

## Conflict of Interest

None declared.
